# Editorial

**Published:** 1903-01

**Authors:** F. E. Daniel


### TEXAS MEDICAL JOURNAL.

AUSTIN, TEXAS.

A MONTHLY JOURNAL OF MEDICINE AND SURGERY.

EDITED AND PUBLISHED BY
F. E. DANIEL, M. D.

ASSOCIATE EDITORS:

WITTEN BOOTH RUSS, M. D., San Antonio, Texas.

P. M. PAYNE, M. D., Brownwood, Texas.

Published Monthly at Austin, Texas. Subscription price $1.00 a year in advance.

Eastern Representative: John Guy Monihan, St. Paul Building, 220 Broadway, New York City.

Official organ of the the West Texas Medical Association, the Houston District Medical Association, the Austin District Medical Society, the Brazos Valley Medical Association, the Galveston County Medical Society, and several others.

I have associated with me in the editorial conduct of the JOURNAL, my son-in-law, Dr. P. M. Payne, of Brownwood, Texas. He will contribute notes on diseases of the eye, ear, nose and throat.—his specialty. My ophthalmological and otological exchanges should be sent to him, and I will thank my colleagues to please note this and change address of exchange copies of all such special publications, to Dr. Payne at Brownwood.

Dr. Russ (San Antonio) is exchange and book editor, and all exchanges except those of eye, ear, nose and throat, and all books for review should be sent to him, except those on Dr. Payne's specialty.

F. E. DANIEL.

